# A Large, Cross-Sectional Observational Study of Serum BDNF, Cognitive Function, and Mild Cognitive Impairment in the Elderly

**DOI:** 10.3389/fnagi.2014.00069

**Published:** 2014-04-15

**Authors:** Hiroyuki Shimada, Hyuma Makizako, Takehiko Doi, Daisuke Yoshida, Kota Tsutsumimoto, Yuya Anan, Kazuki Uemura, Sangyoon Lee, Hyuntae Park, Takao Suzuki

**Affiliations:** ^1^Department of Functioning Activation, Center for Gerontology and Social Science, National Center for Geriatrics and Gerontology, Obu, Japan; ^2^Research Institute, National Center for Geriatrics and Gerontology, Obu, Japan

**Keywords:** brain-derived neurotrophic factor, cognition, biomarker, dementia, aged

## Abstract

**Objective:** The clinical relationship between brain-derived neurotrophic factor (BDNF) and cognitive function or mild cognitive impairment (MCI) is not well-understood. The purpose of this study was to identify the relationship between serum BDNF and cognitive function and MCI, and determine whether serum BDNF level might be a useful biomarker for assessing risk for MCI in older people.

**Materials and Methods:** A total of 4463 individuals aged 65 years or older (mean age 72 years) participating in the study. We measured performance in a battery of neuropsychological and cognitive function tests; serum BDNF concentration.

**Results:** Eight hundred twenty-seven participants (18.8%) had MCI. After adjustment for sex, age, education level, diabetes, and current smoking, serum BDNF was associated with poorer performance in the story memory, and digit symbol substitution task scores. Serum BDNF was marginally associated with the presence of MCI (odds ratio, 95% confidence interval: 1.41, 1.00–1.99) when BDNF was 1.5 SD lower than the mean value standardized for sex and age, education level, diabetes, and current smoking.

**Conclusion:** Low serum BDNF was associated with lower cognitive test scores and MCI. Future prospective studies should establish the discriminative value of serum BDNF for the risk of MCI.

## Introduction

Mild cognitive impairment (MCI) is a transitional condition between normal cognitive function and a clinical diagnosis of probable Alzheimer’s disease (AD). MCI, including amnestic MCI, is a pathologically heterogeneous disorder in which many persons exhibiting mixed pathologies (Schneider et al., 2009). Few studies have investigated biomarkers for MCI. Most work has focused on tau and/or Aβ-42 and their association with neuroimaging results and clinical symptoms in persons at risk for AD. Biomarkers for AD and MCI must be established and validated in larger cohorts, and efforts should be made to investigate markers of other aspects of tau and Aβ pathology, including inflammation and trophic factors (Winblad et al., 2004). Neuronal hypertrophy might constitute an early cellular response to AD pathology or reflect a compensatory mechanism that prevents cognitive impairment despite substantial AD lesions (Riudavets et al., 2007; Iacono et al., 2008, 2009). Neuronal cell growth is modulated by factors such as brain-derived neurotrophic factor (BDNF) (Schindowski et al., 2008). BDNF is highly concentrated in the hippocampus (Phillips et al., 1990), important in synaptic plasticity (Kang and Schuman, 1995; Figurov et al., 1996), and contributes to neurogenesis in the dentate gyrus (Takahashi et al., 1999). BDNF plays a pivotal role in age-related memory impairments and is associated with age-related atrophy of the hippocampus. Previous studies have reported that serum BDNF levels are reduced in AD (Gezen-Ak et al., 2013), MCI (Peng et al., 2005; Yu et al., 2008), major depression disorder, and depressive symptoms (Karege et al., 2002; Shimizu et al., 2003; Cunha et al., 2006; Terracciano et al., 2011). A study of neuronal cell cultures found that amyloid peptide at sublethal concentrations interfered with neuronal plasticity mediated by BDNF signaling cascade (Tong et al., 2004; Wang et al., 2006). Neuronally differentiated P19 mouse embryonic carcinoma cells stimulated by BDNF showed a rapid decrease in tau phosphorylation (Elliott et al., 2005). However, clinical studies that report lower serum BDNF levels are difficult to interpret because of limited knowledge of potential confounders and mixed results based on patient’s age and sex (Bus et al., 2012). Therefore, there is no normal distribution in serum BDNF level, and this may lead to misinterpretation of BDNF levels in studies that used parametric testing with small sample sizes (Ziegenhorn et al., 2007). To establish a cut-off value for serum BDNF is important for clinical purposes, e.g., for helping to increase diagnostic sensitivity. The purpose of this study was to examine the relationships between serum BDNF level and MCI and evaluate whether serum BDNF level may be useful for assessing MCI risk in older adults using a large sample cohort. We explored the relationship between serum BDNF level and MCI, and various measures of cognitive function in elderly adults.

## Materials and Methods

### Study population

**Figure 1:**
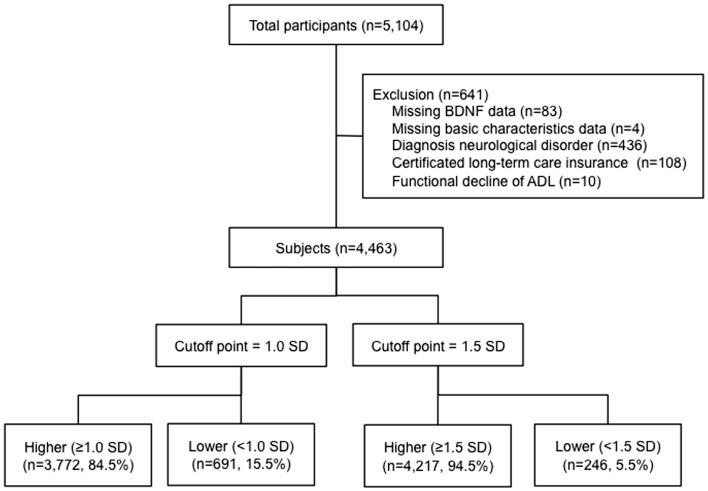
**Flow of participants**. A total of 4463 participants >65 years of age were included in the study. BDNF levels were measured and the participants were divided into those with higher or lower BDNF levels below 1.0 and 1.5 standard deviations from the mean age- and sex-adjusted BDNF value.

Our study assessed 5104 individuals who were enrolled in the Obu study of health promotion for the elderly (OSHPE). Each individual was recruited from Obu, Japan, which is a residential suburb of Nagoya. To be included in this study, each participant was 65 years or older at the time of examination (2011 or 2012), resided in Obu city, and had not participated in another study. We excluded participants who had missing BDNF data and characteristics, diagnosed neurological disorders included stroke, Parkinson’s disease, AD, and depression, certified long-term care insurance, or functional decline of activities of daily living (ADL). Figure 1 shows the flow of participants (Figure 1). Six hundred forty-one of the 5104 participants were excluded and 4463 older adults (range 65–97 years) were included in this study. The data of 4463 individuals were used to analyze in the present study. Informed consent was obtained from all participants prior to their inclusion in the study, and the Ethics Committee of the National Center for Geriatrics and Gerontology approved the study protocol.

### BDNF measurement

Whole blood samples were collected from each patient by venipuncture. To obtain serum, whole blood samples were allowed to coagulate at room temperature (RT) for 30 min and then centrifuged at RT for 15 min at 1000 × *g*. The collected serum was stored in polypropylene tubes at −80°C until assayed. BDNF concentrations were quantitatively determined by enzyme-linked immunosorbent assay (ELISA) using the DuoSet ELISA Development Kit from R&D Systems (Minneapolis, MN, USA). Assays were performed using a specific human BDNF antibody (Minneapolis, MN, USA); no significant cross reactivity or interference was observed in this assay. Serum samples were diluted 1:50. Sample BDNF concentrations were then determined by non-linear regression from the standard curves. Measurements were performed in duplicate and averaged to give a value in picogram per milliliter, which was then expressed in nanogram per milliliter after correcting for sample dilution. “Low” and “High” concentration quality control pools were prepared by adding 10 or 100 ng to 5 ml portions of human serum (Innovative Research, Novi, MI, USA), giving nominal concentrations of 2 and 20 ng/ml, respectively. The assays were performed by one laboratory (SRL Inc., Tokyo, Japan). The repeatability of the BDNF ELISA, as measured by intra-assay precision was 3.8%, and the reproducibility, as measured by inter-assay precision, was 7.6%.

### MCI criteria and cognitive function tests

We defined MCI based on previous studies (Hanninen et al., 2002; Jungwirth et al., 2005; Yaffe et al., 2011), using the following criteria: (1) subjective memory complaints; (2) objective cognitive impairment [indicated by an age-adjusted score at least 1.5 SD below the reference threshold of any of the tests, all of which are commonly used for detailed neuropsychological assessments] but no general cognitive impairment; (3) no evidence of functional dependency (no need for supervision or external help in performing daily activities); and (4) exclusion from the clinical criteria for dementia. Screening for MCI included a standardized personal interview for collecting sociodemographic and lifestyle data, medical history, and functional status (ADL) data, along with cognitive function testing using the mini-mental state examination (MMSE) (Folstein et al., 1975) and the National Center For Geriatrics And Gerontology-Functional Assessment Tool (NCGG-FAT) (Makizako et al., 2012). Individuals who scored ≤23 points on the MMSE were considered to have general cognitive impairment (Anthony et al., 1982). The NCGG-FAT consists of multidimensional cognitive tasks used to assess word-list memory (delayed recall), story memory (delayed recognition), attention and executive function (tablet version of the Trail Making Test – Part A and B), processing speed (tablet version of the symbol digit substitution test), and visuospatial skill (figure selection). The participants were given 20–30 min to complete the battery of tests and their associated tasks. High test–retest reliability and moderate-to-high validity were previously confirmed in community-dwelling older adults for all components of the NCGG-FAT (Makizako et al., 2012). All tests used in this study had previously established standardized thresholds for the definition of cognitive impairment in the corresponding domain (score <1.5 SD below the age-specific mean) for a population-based OSHPE cohort of healthy older adults.

### Potential correlates

Based on the review articles by Bus et al. (2011, 2012), Ziegenhorn et al. (2007), Knaepen et al. (2010), and Plassman (2010), we selected three demographic variables, one physiological variable, two health status indicators, and three behavioral variables as possible confounding factors of the association between BDNF and cognitive decline (Ziegenhorn et al., 2007; Knaepen et al., 2010; Bus et al., 2011, 2012). The three demographic variables – sex, age, and educational level – were selected as possible confounding factors in determining the association of serum BDNF and MCI. Walking speed – the physiological variable – was measured on a flat and straight surface at a comfortable walking speed. Two markers were used to indicate the start and end of a 2.4-m walkway, with a 2-m section to traverse before passing the start marker so that participants were walking at a comfortable pace by the time they reached the timed path. Participants were asked to continue walking for an additional 2 m past the end of the path to ensure a consistent walking pace while on the timed path. Histories of heart disease and diabetes were obtained as health status indicators. Behavioral factors, including current smoking, regular exercise, and frequency of going outdoors, were identified during the interview. Participants were asked whether they currently smoked or exercised regularly: responses were either “yes” or “no.” Participants were asked how often they traveled to places outside their town during a week.

### Statistical analysis

Student’s *t*-test was used to compare BDNF concentrations between men and women. Differences in serum BDNF concentrations were analyzed among four age-groups (65–69, 70–74, 75–79, 80–84, and ≥85 years) by one-way analysis of variance (ANOVA) in both sexes. A linear regression was used to analyze the relationships between BDNF concentration and age and education in both sexes. Participants were divided into two groups according to 1.0 or 1.5 SD from age- and sex-specific mean values among the four age-groups (Figure 1). Independent sample *t*-tests or Chi-square tests were used to compare the potential correlates and cognitive performance between: (a) participants who had BDNF levels below 1.0 SD and above 1.0 SD; and (b) participants who had BDNF levels below 1.5 SD and above 1.5 SD. Linear regression analyses (forced-entry) were used to reveal the relationships between BDNF concentration and cognitive performance. Multivariate logistic regression analyses, forced-entry, were used to determine adjusted odds ratios (ORs) and 95% confidence intervals (95% CIs), and to assess independent associations between the serum BDNF levels and MCI. The covariates of sex, age, and educational level, and significant variables in univariate analyses were added to the regression models to evaluate independent associations between BDNF and cognitive performances or MCI. Logistic regression models determined the crude OR and the adjusted OR of BDNF for 1.0 and 1.5 SD. Sensitivity, specificity, and positive and negative likelihood ratios of the BDNF values with MCI were calculated. We excluded the participants who scored ≤23 points on the MMSE and did not complain of memory loss. We used the data of MCI (*n* = 827) and cognitive healthy (*n* = 2533) elderly adults in the logistic regression analyses. All statistical comparisons were made at the 0.05 level of significance, and all data management and statistical computations were performed using the IBM SPSS Statistics 20.0 software package (SPSS Inc., Chicago, IL, USA).

## Results

**Figure 2:**
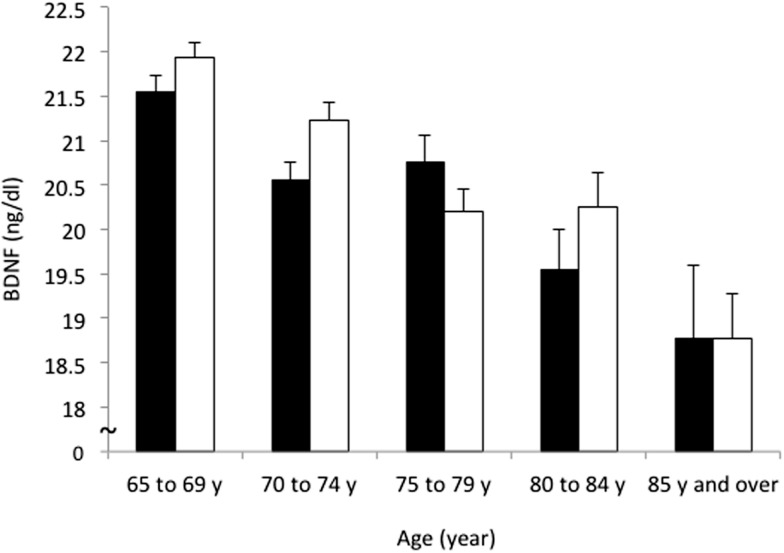
**Sex and age differences in serum BDNF concentration**. Mean and standard error of serum BDNF levels are shown for each 5-year increment in age. Serum BDNF decreased with aging in men (black bars) and women (white bars; *P* < 0.001) and women showed higher BDNF levels than men (*P* = 0.031).

The mean BDNF concentrations were statistically significantly different in men (20.8 ± 5.6 ng/ml) and women (21.2 ± 5.2 ng/ml; *t* = 2.162, *df* = 4394, *P* = 0.031). BDNF concentrations declined with increasing age in both sexes (*F* = 24.822, *df* = 3, *P* < 0.001) (Table 1; Figure 2). Linear regression found that serum BDNF was associated with age in men (β = −0.123, *t* = −5.750, *P* < 0.001) and women (β = −0.154, *t* = −7.475, *P* < 0.001). Education level was associated with serum BDNF in women (β = 0.045, *t* = 2.149, *P* = 0.032), but not in men (β = 0.012, *t* = 0.564, *P* = 0.573).

**Table 1:** **Serum BDNF levels among the four age-groups**.

	Men		Women	
	BDNF values 1.0 SD lower than the mean	BDNF values 1.5 SD lower than the mean	BDNF values 1.0 SD lower than the mean	BDNF values 1.5 SD lower than the mean
65–69 years	16.08	13.34	16.75	14.15
70–74 years	15.20	12.52	15.85	13.16
75–79 years	14.82	11.84	15.12	12.57
80 years and over	13.30	10.27	15.05	12.63

The comparison between participants who had BDNF levels below 1.0 SD and above 1.0 SD, revealed that the participants below 1.0 SD had a higher prevalence of diabetes, a lower proportion of smokers, higher scores of story memory, and a symbol digit substitution task, compared with participants who had BDNF levels above 1.0 SD. The results were similar for the comparison between the participants who had BDNF levels below 1.5 SD and above 1.5 SD. A comparison of MCI prevalence found no significant difference between the participants who had serum BDNF below and above 1.0 SD. In contrast, when serum BDNF was dichotomized according to 1.5 SD below the mean, a significant difference was found in MCI (Table 2). The mean BDNF concentrations did not show significant differences between MCI participants (20.9 ± 5.3 ng/ml) and non-MCI participants (21.2 ± 5.4 ng/ml; *t* = 1.362, *df* = 3358, *P* = 0.173).

**Table 2:** **Comparisons between BDNF levels of 1.0 and 1.5 SD from the mean**.

	BDNF levels of 1.0 SD from the mean			BDNF levels of 1.5 SD from the mean		
	Participants above 1.0 SD	Participants below 1.0 SD	*P*	Participants above 1.0 SD	Participants below 1.0 SD	*P*
Sex, women, *n*, %	1919, 50.9	372, 53.8	0.152	2175, 51.6	116, 47.2	0.177
Age, years	71.9 ± 5.4	72.1 ± 5.5	0.395	71.9 ± 5.5	71.8 ± 5.2	0.744
Education level, years, 10^a^	11.4 ± 2.5	11.3 ± 2.5	0.294	11.4 ± 2.5	11.2 ± 2.5	0.237
Walking speed, m/s, 6^a^	1.3 ± 0.2	1.3 ± 0.2	0.236	1.3 ± 0.2	1.3 ± 0.2	0.722
Heart disease, yes, *n*, 2^a^	585, 15.5	124, 17.9	0.109	671, 15.9	38, 15.4	0.844
Diabetes, yes, *n*	474, 12.6	111, 16.1	0.012	538, 12.8	47, 19.1	0.004
Current smoking, yes, *n*, %, 1^a^	392, 10.4	51, 7.4	0.015	430, 10.2	13, 5.3	0.012
Habitual exercise, yes, *n*, 5^a^	2816, 74.8	519, 75.1	0.844	3152, 74.8	183, 74.4	0.876
Going outdoors, times/week, 1^a^	5.9 ± 1.6	5.8 ± 1.7	0.125	5.9 ± 1.7	5.8 ± 1.7	0.841
MMSE score, 6^a^	26.3 ± 2.7	26.2 ± 2.8	0.64	26.3 ± 2.7	26.0 ± 2.8	0.09
Word-list memory score, 19^a^	3.8 ± 2.0	3.8 ± 2.0	0.872	3.8 ± 2.0	3.7 ± 2.0	0.466
Story memory score, 26^a^	6.8 ± 1.9	6.6 ± 1.9	0.029	6.7 ± 1.9	6.4 ± 1.9	0.011
Trail making test – part A, s, 11^a^	21.2 ± 6.9	21.5 ± 7.3	0.261	21.2 ± 7.0	22.0 ± 7.1	0.083
Trail making test – part B, s, 15^a^	43.1 ± 17.9	44.1 ± 18.4	0.173	43.2 ± 17.9	45.3 ± 18.7	0.068
Symbol digit substitution task, 14^a^	38.4 ± 8.4	37.5 ± 8.5	0.013	38.3 ± 8.4	37.2 ± 8.4	0.049
Visuospatial skill score, 85^a^	5.2 ± 1.5	5.2 ± 1.5	0.928	5.2 ± 1.5	5.2 ± 1.4	0.798
Mild cognitive impairment, yes, *n*, %, 73^a^	689, 24.2	138, 24.6	0.244	774, 24.3	53, 31.0	0.047

*^a^Number of missing data*.

Table 3 shows the association between serum BDNF and performance on various cognitive function tests using multiple linear regression, adjusted for sex, age, education level, diabetes, and current smoking status. Serum BDNF levels were associated with a decline in story memory (β = 0.027, *t* = 1.958, *P* < 0.05) and digit symbol substitution test scores (β = 0.027, *t* = 2.172, *P* < 0.05). There was no significance between BDNF and MMSE for word-list memory, the tablet version of the Trail Making Test – Part A and B, or figure selection.

**Table 3:** **Multiple linier regression analyses with serum BDNF, potential confounders, and cognitive tests**.

Independent variable	Dependent variables													
	MMSE		Word-list memory		Story memory		Trail making test – part A		Trail making test – part B		Symbol digit substitution task		Visuospatial skill	
	β	*P*	β	*P*	β	*P*	β	*P*	β	*P*	β	*P*	β	*P*
BDNF, ng/ml	0.011	0.442	0.017	0.229	0.027	0.050	− 0.008	0.584	− 0.022	0.091	0.027	0.030	− 0.011	0.472
Sex, men = 1, women = 2	0.156	<0.001	0.160	<0.001	0.107	<0.001	− 0.032	0.027	− 0.027	0.050	− 0.028	0.030	− 0.074	<0.001
Age, years	− 0.208	<0.001	− 0.316	<0.001	− 0.322	<0.001	0.354	<0.001	0.400	<0.001	− 0.473	<0.001	− 0.167	<0.001
Education, years	0.219	<0.001	0.176	<0.001	0.242	<0.001	− 0.167	<0.001	− 0.235	<0.001	0.230	<0.001	0.192	<0.001
Diabetes, no = 1, yes = 2	0.009	0.512	− 0.016	0.253	− 0.015	0.254	0.023	0.091	0.006	0.616	− 0.036	0.003	− 0.015	0.306
Current smoking, no = 1, yes = 2	− 0.042	0.004	− 0.009	0.522	0.004	0.781	0.031	0.029	0.057	<0.001	− 0.059	<0.001	0.012	0.421

In all, 827 participants (18.8%) had MCI. A total of 691 participants (15.5%) had BDNF levels below 1.0 SD from the mean, and 246 participants (5.5%) had levels below 1.5 SD from the mean. Table 4 shows the association between serum BDNF levels and the diagnosis of MCI using multiple logistic regression, adjusted for sex, age, education level, diabetes, and current smoking status. The crude logistic model showed significant relationships between MCI and BDNF: 1.5 SD (OR, 1.40; 95% CI, 1.00–1.96), age (OR, 1.02; 95% CI, 1.01–1.04), and education (OR, 0.82; 95% CI, 0.79–0.85). The adjusted logistic model for BDNF 1.0 SD showed no significant relationship between serum BDNF and MCI. In contrast, when serum BDNF was dichotomized according to 1.5 SD below the mean, a significant association with MCI was found (OR, 1.41; 95% CI, 1.00–1.98). Education was also associated with MCI (OR, 0.82; 95% CI, 0.79–0.85). Sensitivity and specificity of the BDNF values for 1.5 SD were 6.4% (95% CI: 4.8–8.3%) and 95.3% (95% CI: 94.5–96.1%), respectively. Positive and negative likelihood ratios of the BDNF values of 1.5 SD were 1.38 (95% CI: 1.00–1.88) and 0.98 (0.96–1.00), respectively.

**Table 4:** **Relationships between MCI and BDNF or selected correlates**.

	Crude OR		Adjusted OR in BDNF 1.0 SD		Adjusted OR in BDNF 1.5 SD	
	OR (95% CI)	*P*	OR (95% CI)	*P*	OR (95% CI)	*P*
BDNF 1.0 SD, below/above	1.14 (0.92–1.40)	0.244	1.14 (0.92–1.42)	0.236		
BDNF 1.5 SD, below/above	1.40 (1.00–1.96)	0.048			1.41 (1.00–1.98)	0.050
Sex, women/men	1.00 (0.86–1.17)	0.971	0.85 (0.71–1.00)	0.051	0.85 (0.72–1.01)	0.063
Age, years	1.02 (1.01–1.04)	0.003	1.00 (0.99–1.02)	0.977	1.00 (0.99–1.02)	0.942
Education, years	0.82 (0.79–0.85)	<0.001	0.82 (0.79–0.85)	<0.001	0.82 (0.79–0.85)	<0.001
Diabetes, yes/no	1.11 (0.88–1.39)	0.377	1.04 (0.82–1.31)	0.752	1.03 (0.82–1.31)	0.778
Current smoking, yes/no	1.09 (0.84–1.43)	0.517	1.19 (0.90–1.59)	0.23	1.20 (0.90–1.60)	0.208

## Discussion

In our cross-sectional observational study of 4463 community-living older adults, serum BDNF was associated with a decline in story memory and digit symbol substitution test scores, even when adjusted for sex, age, education, diabetes, and current smoking. Moreover, serum BDNF levels of 1.5 SD lower than the age- and sex-adjusted means were associated with a significant risk of MCI. These results suggest that serum BDNF may be a useful biomarker of cognitive function and MCI status in the elderly.

In demographic variables, serum BDNF was higher in women than men. Similar results were found by Trajkovska et al. (2007) using both serum and whole blood BDNF, whereas they were in contrast to other studies using only serum BDNF (Lang et al., 2004; Ziegenhorn et al., 2007). Another study found a significant interaction of age and menopausal state with BDNF in women, with age-related increases serum BDNF premenopause and age-related decreases postmenopause (Bus et al., 2011). Estrogen levels are significantly associated with BDNF levels (Scharfman and MacLusky, 2006), so the postmenopausal drop in estrogen could result in decreased serum BDNF. Therefore, the differences in serum BDNF levels in men and women might be related to sex hormone differences. However, it is difficult to draw conclusions with cross-sectional approaches, and longitudinal studies are needed.

Among lifestyle measures, diabetes and current smoking showed significant differences between the participants who had high and low serum BDNF levels. Low levels of BDNF accompanied impaired glucose metabolism. Krabbe et al. reported that plasma levels of BDNF were decreased in participants with type 2 diabetes independent of obesity (Krabbe et al., 2007). Plasma BDNF was inversely associated with fasting plasma glucose, but not with insulin. When plasma insulin was increased while maintaining normal blood glucose, the cerebral output of BDNF was not inhibited, indicating that high levels of glucose, but not insulin, inhibit the output of BDNF from the human brain. They concluded that the cerebral output of BDNF, which is negatively related to high plasma glucose levels and decreased BDNF, may be a pathogenetic factor involved not only in dementia, but also in type 2 diabetes. The results of our cohort support previous findings. Smoking was associated with higher BDNF levels; this finding is consistent with several studies. In animal studies, regional brain BDNF expression was altered by exposure to or withdrawal from nicotine (Kenny et al., 2000). In some human studies, smoking cessation increased BDNF serum levels over the span of several months (Kim et al., 2007; Bhang et al., 2010). A recent epidemiological study of 1168 subjects aged 18–65 years also reported an independent relationship between smoking and serum BDNF levels, with higher BDNF in former and current smokers compared to subjects who never smoked (Bus et al., 2011). The results of our study confirm this relationship between BDNF and smoking in adults 65 years of age and older. Nicotine has induced SH-SY5Y neuroblastoma cell proliferation through BDNF and its receptor, TrkB. The activation of nicotinic receptors has effects upon the BDNF–TrkB pathway, inducing cell proliferation by promoting the release of BDNF, which in turn activates TrkB receptors (Serres and Carney, 2006). Moreover, the beta-arrestin-2 protein is important in induction and expression of nicotine sensitization as well as nicotine’s effects on accumbal BDNF (Correll et al., 2009).

Brain-derived neurotrophic factor is highly concentrated in the hippocampus (Phillips et al., 1990; Wetmore et al., 1990). A single nucleotide polymorphism in the BDNF gene affects the regulated secretion of BDNF in the hippocampus (Egan et al., 2003) and has been related to lower serum levels of BDNF (Ozan et al., 2010) and smaller hippocampal volumes (Pezawas et al., 2004; Szeszko et al., 2005), which can lead to deficits in executive function (Frodl et al., 2006) and memory function (Erickson et al., 2009). The hippocampus–orbitomedial prefrontal circuit integrates cognition, emotion, and behavior, thereby influencing working memory and executive functions (Wall and Messier, 2001). The observed relationship between lower serum BDNF and impaired memory and processing speed is consistent with previous studies. However, the relationships between serum BDNF and executive function, the Trail Making Test – Part B, did not reach significance (*P* = 0.09). Further studies will be needed to establish the relationships between serum BDNF and executive function in the elderly adults.

Serum BDNF values 1.5 SD lower than the age- and sex-adjusted mean were associated with MCI, whereas serum BDNF levels lower than 1.0 SD from age- and sex-adjusted mean serum BDNF values were not. These results suggest that the participants who had 1.5 SD lower than the mean age- and sex-adjusted BDNF values may pose a risk of cognitive impairment. BDNF supports cholinergic, dopaminergic, serotonergic, and neuropeptide-containing neurons (Hyman et al., 1991; Knusel et al., 1991; Mamounas et al., 1995) and may play an important role in AD-related pathophysiology. Animal studies found that Aβ disrupts BDNF signaling and that BDNF protects against Aβ toxicity via TrkB signaling (Tapia-Arancibia et al., 2008). Lower levels of both BDNF and TrkB have been found in postmortem brains of individuals with AD (Murer et al., 2001). BDNF levels are significantly reduced in the hippocampus and parietal cortex and BDNF/neurotrophin 3 ratios are lower in frontal and parietal cortices in patients with AD compared with age-matched controls (Hock et al., 2000). Higher serum levels of BDNF in individuals with AD are predictive of slower rates of decline (Laske et al., 2011). Peng et al. (2005) reported strong relationships between MMSE and Global Cognitive Score results and proBDNF and mature BDNF levels. Decreased serum BDNF in the preclinical stages of AD further suggests that BDNF and proBDNF deficiency play a pivotal role in cell atrophy, cell loss, and synaptic dysfunction, with a lack of trophic support contributing to the degeneration of specific neuronal subpopulations in the AD-affected brain (Hock et al., 2000; Laske et al., 2007).

Other studies have shown that BDNF serum levels increase in MCI and AD patients (Angelucci et al., 2010). This increase may reflect a compensatory repair mechanism in early and late neurodegeneration that is protective by contributing to Aβ degradation. Laske et al. (2006) found that patients in the early stages of probable AD with MMSE scores ≥21 (mean of 25.5) had significantly higher serum BDNF levels compared to patients in late-stage AD with MMSE scores <21 (mean of 13.3) and age-matched healthy controls. The study also showed a tendency toward lower BDNF levels in patients with late-stage AD and progressive dementia (mean MMSE: 13.3; range: 6–20). The mean MMSE scores of our MCI participants was 26.6 (range: 24–30), higher than that of patients in the early stages of probable AD in the Laske et al. (2006) study. Our MCI participants may have been at a stage earlier than the point at which the BDNF compensatory repair mechanism is triggered in early neurodegeneration.

The strengths of the present study include the large sample size and comprehensive measurement of cognitive function, which correlates closely with dementia. One limitation of the study is that the analysis is based on cross-sectional data. Although our study was population-based, further prospective investigations are needed to validate using 1.5 SD serum BDNF levels for discriminating the risk of cognitive decline and MCI in older people. Sensitivity of the 1.5 SD serum BDNF levels to discriminate MCI and healthy participants showed a very low value (6.4%). The result suggests that it is necessary to review the discrimination point to screen MCI in the community with high sensitivity. BDNF is reduced in elderly individuals with major depression and bipolar disorder, with distinct dynamics according to the disease stages, treatment, or the presence of cognitive impairment (Molendijk et al., 2011; McKinney and Sibille, 2013; Sibille, 2013). In a recently published study, Diniz et al. (2014) showed a significant decline in serum BDNF level over 2 years of follow-up only in those individuals with persistent cognitive decline (Diniz et al., 2014). Therefore, BDNF seems to be a non-specific marker for many neuropsychiatric disorders, thus, reducing its discriminative power to identify individuals with MCI. Our study also excluded older adults with neurological disorders and those adults who were certified for long-term care insurance due to functional decline. Therefore, the study findings may not be generalized to these patient groups. It is likely that several clinical and etiological heterogeneities exist between subtypes of MCI (Petersen, 2004). Although amnestic MCI appears to be most closely linked with AD, there are many concomitant pathologic abnormalities, including argyrophilic grain disease, hippocampal sclerosis, and vascular lesions (Petersen et al., 2006). The findings of the study about the relationships between serum BDNF and MCI may change in further analyses of each subtype of MCI.

In conclusion, we provide preliminary evidence that serum BDNF can be associated with lower cognitive test scores in older people. In our cohort, serum BDNF was marginally associated with the presence of MCI when BDNF was 1.5 SD lower than the mean age- and sex-adjusted values. Future prospective studies should establish the discriminative value of serum BDNF for a risk of MCI and its validity as a screening test for this population.

## Author Contributions

Study concept and design: Hiroyuki Shimada, Takao Suzuki; acquisition of data: Hyuma Makizako, Takehiko Doi, Daisuke Yoshida, Kota Tsutsumimoto, Yuya Anan, Kazuki Uemura; analysis and interpretation of data: Sangyoon Lee; critical revision of the manuscript: Hyuma Makizako, Hyuntae Park; statistical analysis: Hyuntae Park; drafting of the manuscript: Hiroyuki Shimada; obtaining funding: Hiroyuki Shimada, Takao Suzuki, Hyuma Makizako; study supervision: Takao Suzuki.

## Conflict of Interest Statement

The authors declare that the research was conducted in the absence of any commercial or financial relationships that could be construed as a potential conflict of interest.
